# Mechanical Thrombectomy for Pulmonary Tumor Embolism Masquerading as Bland Pulmonary Embolism: A Case Report

**DOI:** 10.7759/cureus.117346

**Published:** 2026-10-02

**Authors:** Vritika Bhardwaj, Rohit Agarwal, Gaurav Chauhan, Alok Gupta

**Affiliations:** 1 Department of Interventional Radiology, Medanta Hospital, Lucknow, IND; 2 Department of Medical Oncology, Max Super Speciality Hospital, Lucknow, IND

**Keywords:** large bore aspiration thrombectomy, percutaneous aspiration thrombectomy, pulmonary embolism (pe), pulmonary thrombectomy, pulmonary tumor embolism

## Abstract

Pulmonary tumor embolism (PTE) is a rare but potentially life-threatening form of non-thrombotic pulmonary embolism. Despite advancements in management, it remains a challenging entity due to its unpredictable clinical course and high mortality rate. With the advent of minimally invasive endovascular treatment modalities, the management of pulmonary embolism has witnessed a shift from systemic thrombolysis or surgical thromboarterectomy to catheter-directed thrombolysis, mechanical thrombectomy, or pharmaco-mechanical thrombectomy, combining mechanical thrombectomy with catheter-directed thrombolysis. We report a rare case of intermediate-risk pulmonary tumor embolism managed by large-bore aspiration thrombectomy.

## Introduction

Pulmonary tumor embolism (PTE) refers to a rare entity characterized by dislodgement of a primary or metastatic neoplasm, embolized and impacted into the pulmonary vasculature, resulting in vessel occlusion and cardiovascular compromise. PTE most commonly occurs in patients with advanced malignancies, particularly adenocarcinomas, and can manifest as acute or subacute respiratory distress. It must be considered a possibility in patients with an underlying neoplasm. The embolus might be tumoral, mycotic, bacterial, fat, or amniotic in composition [1,2]. The incidence of tumoral embolism ranges from 0.3% to 26% in patients with solid malignancy and is more commonly seen in patients with mucin-producing tumors like the primary malignancies of the colon, lung, breast, and stomach, or in cases of sarcoma [3,4].

Despite advancements in management, PTE remains challenging due to its unpredictable clinical course and high mortality rate [5]. It is characterized by the migration of tumor cells or tumor-derived emboli to the pulmonary vasculature, resulting in acute or subacute respiratory compromise. While venous thromboembolism (VTE) is a well-recognized complication in cancer patients, PTE represents a distinct entity with unique clinical features, diagnostic challenges, and therapeutic considerations [3]. Histopathological analysis of pulmonary emboli in PTE often reveals tumor cells within the thrombus, confirming the neoplastic origin of the embolic material [5]. Herein, we report a rare case of intermediate-risk pulmonary embolism managed by large-bore aspiration thrombectomy in a patient with adenocarcinoma of the colon, resulting in significant improvement in the patient's clinical condition.

## Case presentation

A 65-year-old female with adenocarcinoma of the colon post-extended right hemicolectomy presented to the emergency department with worsening exertional dyspnea despite being on therapeutic anticoagulation (Rivaroxaban, 20 mg once daily). Biochemical evaluation showed raised D-dimer (920 ng/mL), hs-troponin I (35.14 ng/L), and N-terminal pro-brain natriuretic peptide (NT-proBNP) levels (1210 pg/mL) (Table 1).

**Table 1:** Biochemical parameters with patient values and corresponding normal reference range NTproBNP: N-terminal pro-brain natriuretic peptide

Biochemical parameters	Patient value	Normal reference range
D-dimer	920 ng/mL	<500 ng/mL
hs-troponin I	35.14 ng/L	<15.7 ng/L (female)
NTproBNP	1210 pg/mL	<125 pg/mL (for age less than 75 years)

CT pulmonary angiography showed diffuse multifocal intraluminal hypodense filling defects involving the left pulmonary trunk and involving all the distal segmental branches of the upper and lower lobes without any contrast enhancement. Poor opacification of the left pulmonary vein and its branches was also noted (Figure 1). The right-to-left ventricle (RV/LV) ratio was also calculated and found to be 1. The cardiac biomarkers and RV/LV ratio indicated the right ventricular strain resulting from pulmonary embolism, putting the patient in the intermediate-risk category.

**Figure 1:**
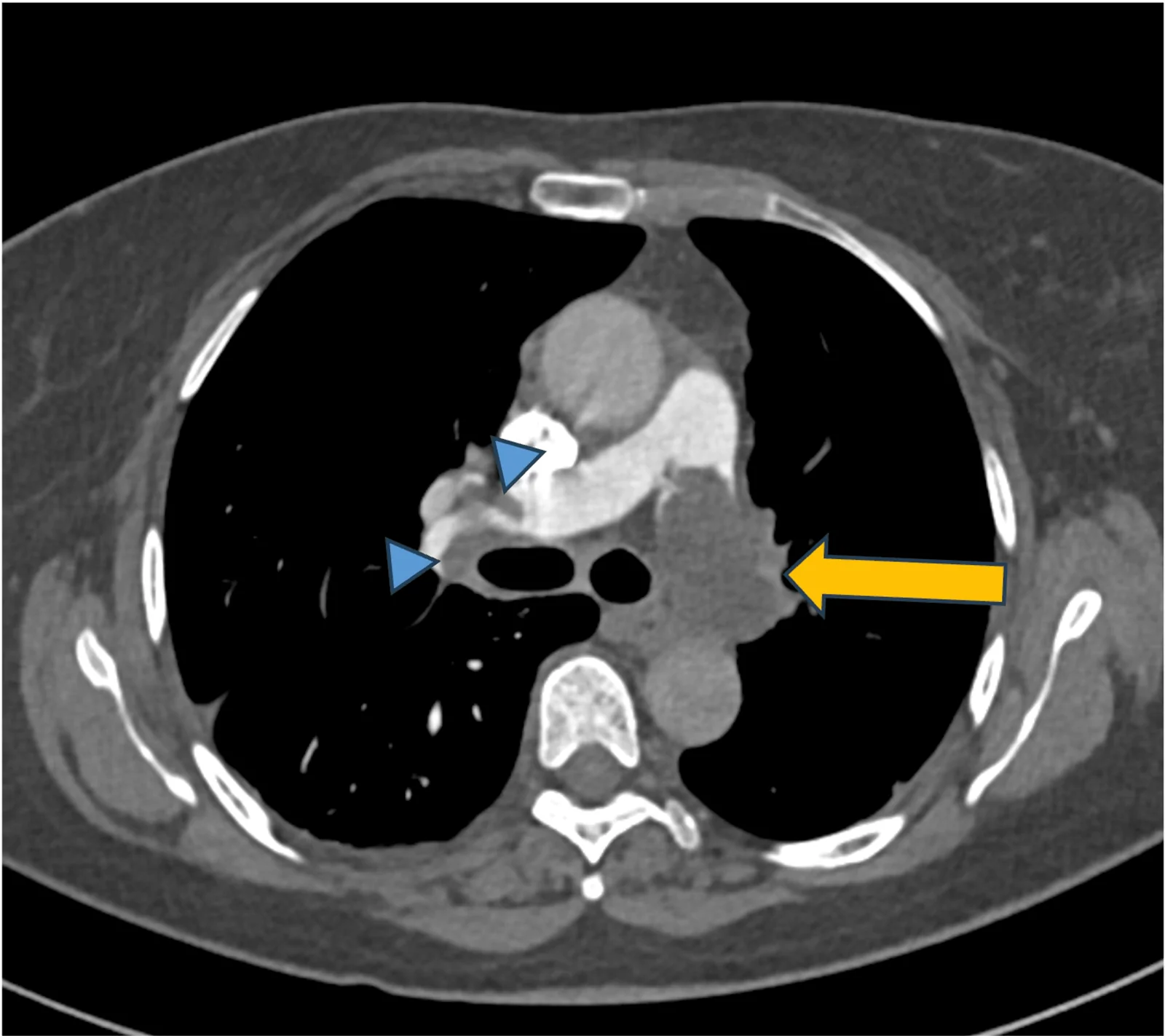
CT pulmonary angiogram showing acute pulmonary thrombosis Axial sections showing non-enhancing hypoattenuating eccentric thrombus in right pulmonary arterial branches (blue arrowheads) and complete thrombosis in left branch of pulmonary artery (yellow arrow)

Considering the progressive clinical deterioration despite therapeutic anticoagulation, the case was discussed with the Pulmonary Embolism Response Team (PERT), and mechanical thrombectomy was planned after obtaining informed consent.

Under aseptic conditions and using ultrasound guidance, access was obtained into the right common femoral vein by the Seldinger technique, and a 16F long sheath (45 cm, Check-Flo Performer, Cook Medical, Bloomington, Indiana, USA) was inserted. Subsequently, a 5F PICARD catheter (100 cm, Cook Medical) was advanced into the right atrium over a floppy-tip guidewire (Glidewire, Terumo, Somerset, NJ, USA). The sheath was advanced over the catheter-wire assembly with the tip positioned at the cavo-atrial junction. Catheter-wire assembly was negotiated through the right ventricle into the main pulmonary artery, and an angiogram was taken, which confirmed the findings of CT pulmonary angiography (Figure 2).

**Figure 2:**
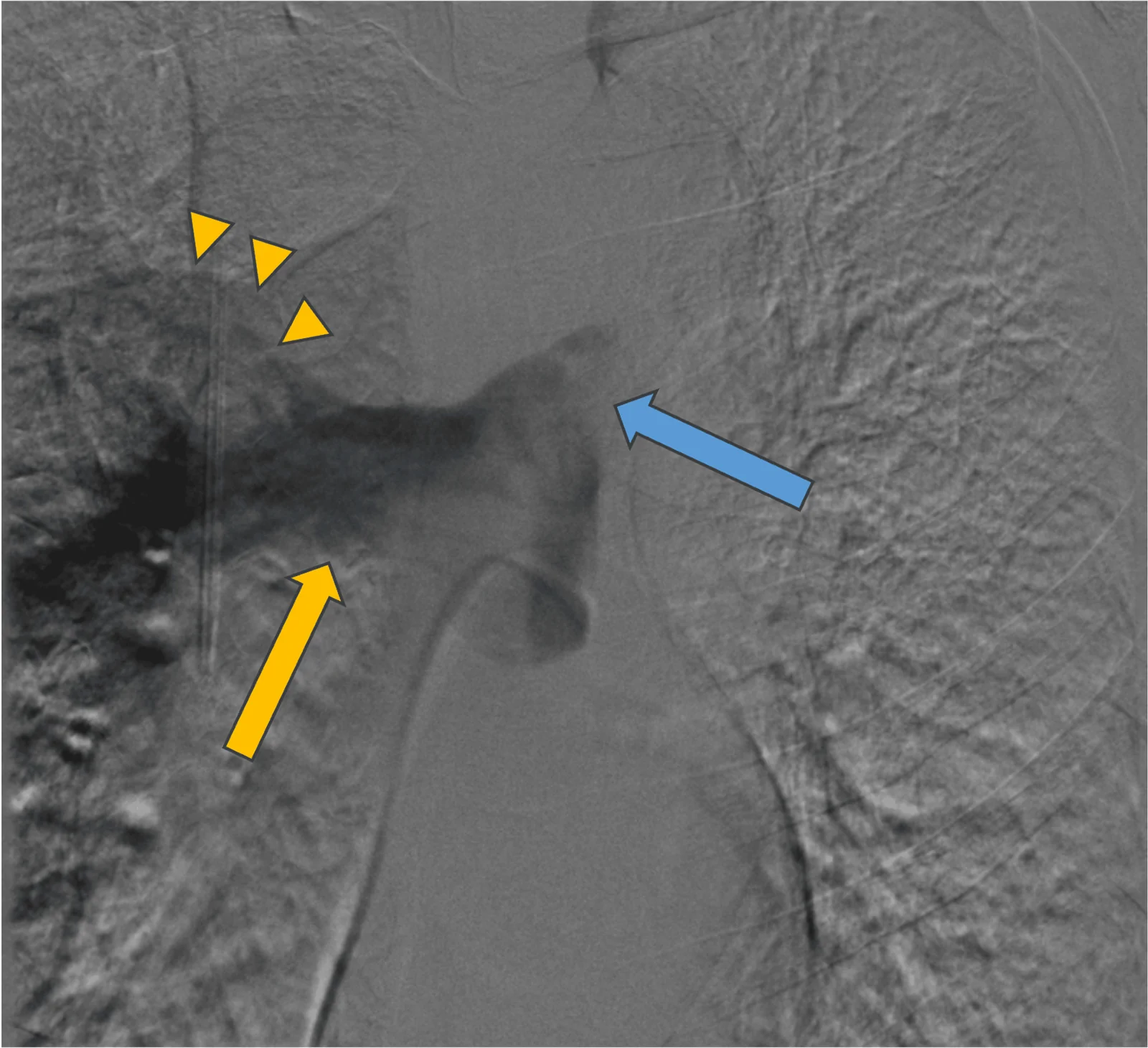
Pre-thrombectomy pulmonary angiogram Pre-thrombectomy pulmonary angiogram showing a cut-off and filling defect in the left branch of pulmonary artery (blue solid arrow) without any opacification of intrapulmonary vessels. Normal contrast opacification of right branch of pulmonary artery (yellow solid arrow) and interlobar artery is noted with attenuation of upper lobe segmental branch (yellow arrowheads).

Over the wire, computer-assisted vacuum thrombectomy (CAVT) was done with the Indigo System CAT 16 catheter with HTORQ tip (Penumbra, Inc., Alameda, CA, USA) and Lightning-12 Intelligent Aspiration system (Penumbra, Inc.). The CAT 16 catheter was advanced into the pulmonary arteries, and multiple passes were taken. A large thrombus fragment was removed in the initial pass from the left main pulmonary artery; however, later, a few passes were ineffective. The post-thrombectomy angiogram showed adequate recanalization of the left main pulmonary trunk; however, distal opacification and parenchymal perfusion were not adequate initially (Figure 3). Subsequently, aspiration thrombectomy was done with the catheter advanced further into the interlobar left pulmonary arterial branches. Final angiograms showed decent recanalization with pulmonary parenchymal perfusion. Total blood loss during the procedure was 200 mL, fluoroscopic time was 17.2 minutes, and about 120 mL of diluted contrast (50% Omnipaque) was administered.

**Figure 3:**
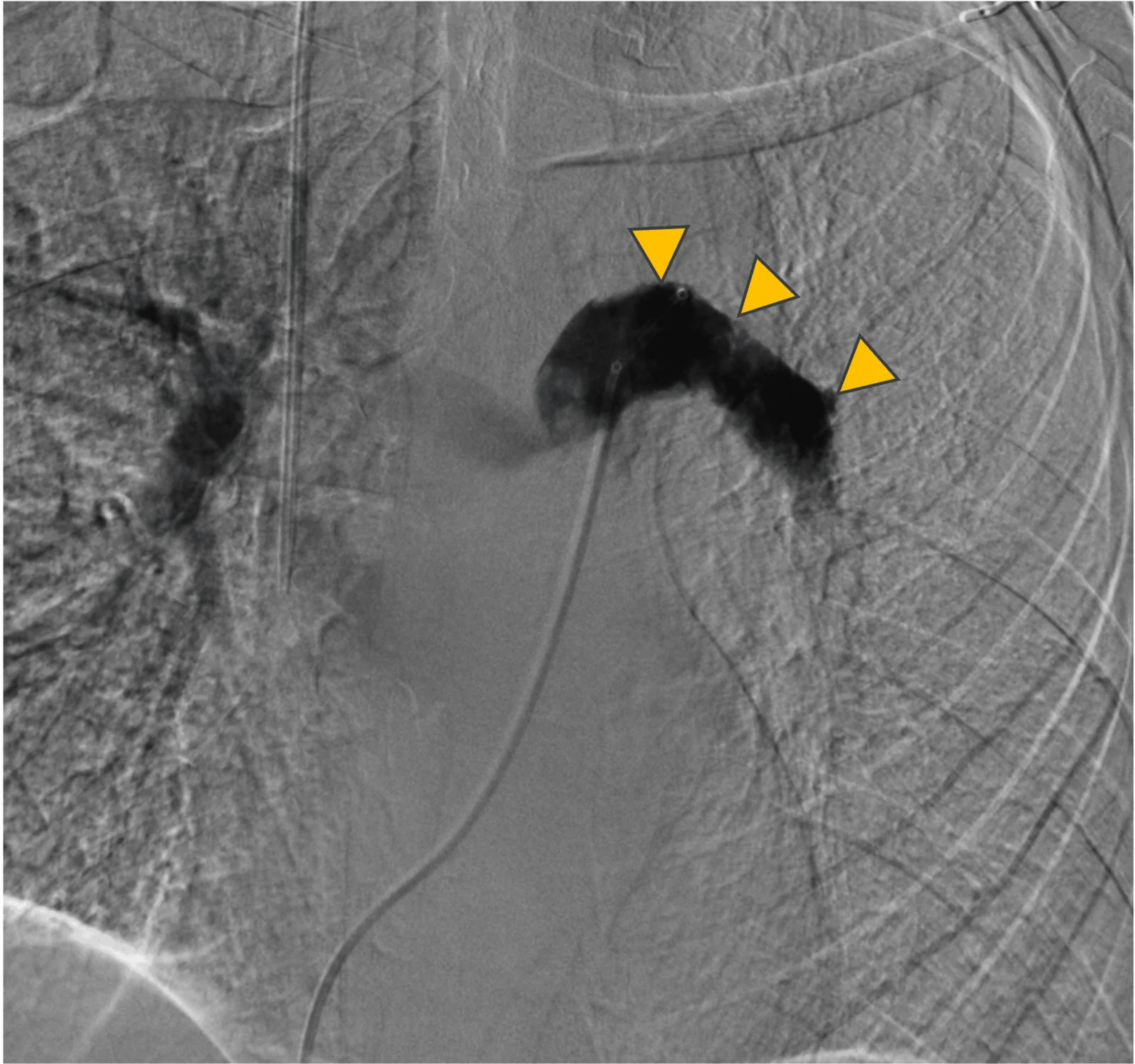
Post-thrombectomy angiography Post-thrombectomy digital subtraction angiography (DSA) image showed recanalization initially with stasis in the left branch of pulmonary artery and ipsilateral interlobar artery (yellow arrowheads) which could be due to the residual mucin content resulting from the tumoral thrombus.

The aspirated clot was examined, and the tumoral nature of the pulmonary embolism was suspected (Figure 4).

**Figure 4:**
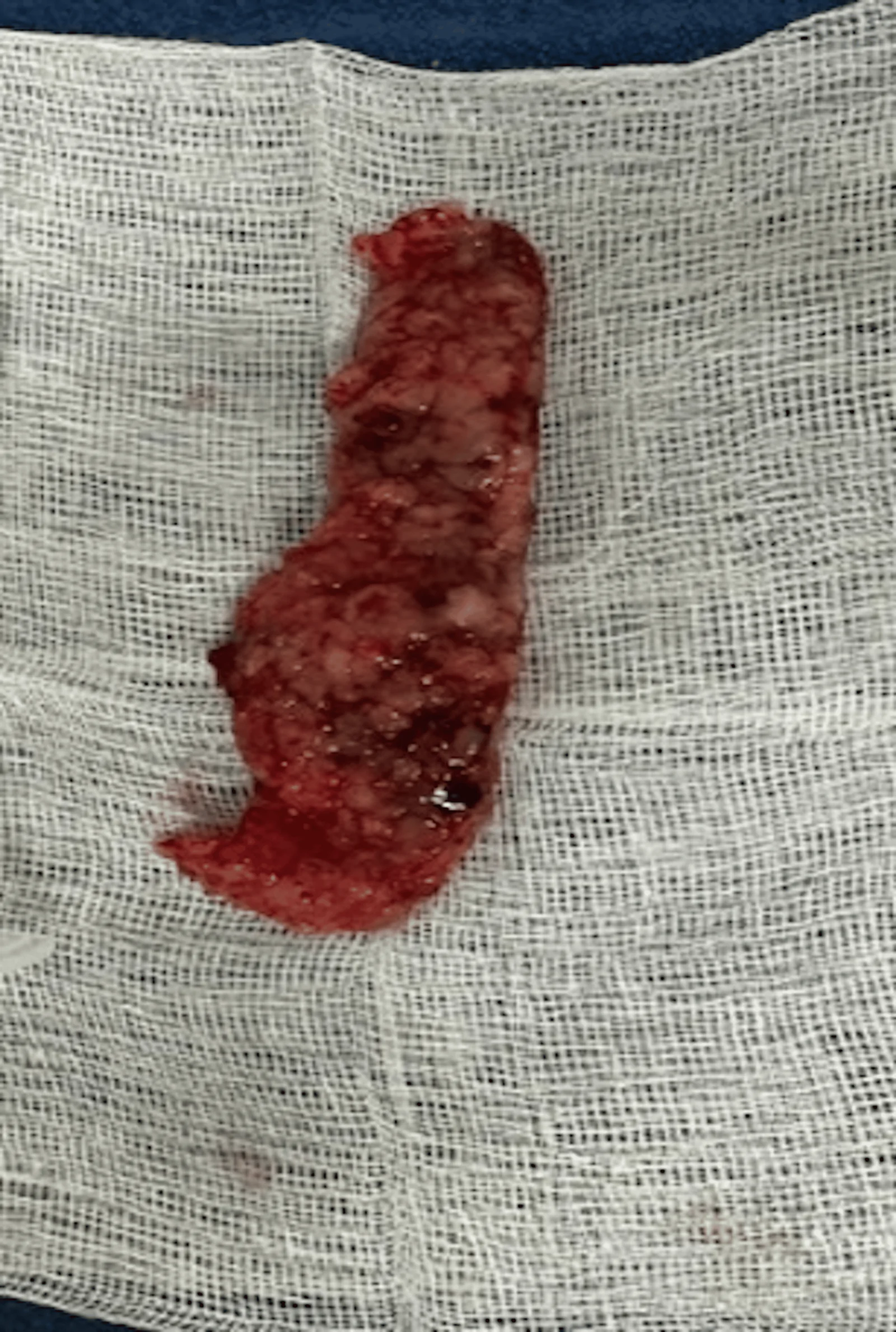
Gross specimen of thrombus Gross specimen of the thrombus aspirated post mechanical thrombectomy containing a soft-tissue component with hemorrhagic/necrotic areas. The histopathological evaluation of the thrombus confirmed its metastatic nature.

The sample was sent for histopathological evaluation. Subsequently, the access sheath was removed, and hemostasis was achieved by the “figure of eight” suturing technique followed by manual compression.

The intervention was technically as well as clinically successful, as the patient experienced significant improvement in vital parameters and a decrease in oxygen requirements over the next few days. Eventually, histopathological analysis showed the specimen as a tumoral thrombus containing metastatic adenocarcinoma with extracellular mucin. At the one-week follow-up, the patient showed significant improvement in dyspnea while being on therapeutic anticoagulation (Rivaroxaban, 20 mg once daily) and medical management as per the oncology team. Six months later, the patient presented to the interventional radiology clinic without any new episode of dyspnoea or hypotension.

## Discussion

Clinical presentation

The clinical presentation of PTE is highly variable, ranging from incidental asymptomatic findings to life-threatening hemodynamic instability and death [6]. Usually, PTE due to sarcoma presents in the acute phase, while cases of non-sarcoma tumoral embolism present in the subacute phase [7,8]. Although a clear-cut demarcation between the clinical findings of tumoral and non-tumoral pulmonary embolism is difficult, subacute dyspnea in a patient with known neoplasm should raise clinical suspicion of tumoral thrombus.

Sequelae

Untreated PTE may resolve, remain stable and asymptomatic, or show progression [9]. Progression of the disease can lead to an increase in pulmonary vascular resistance, which subsequently can lead to intimal fibrosis and proliferation of smooth muscle cells in the distal pulmonary arteries. Eventually, pulmonary hypertension ensues, and the increased afterload on the right ventricle leads to cor pulmonale. Although cor pulmonale develops in less than half of the patients with pulmonary hypertension, it significantly increases the mortality rate in patients with PTE [2,10].

Diagnosis

The preferred initial imaging modality for the diagnosis of PTE is CT pulmonary angiography [6,11]. Imaging features like hypo-attenuating filling defects, beaded pulmonary arteries, enhancing filling defects, and progressive pulmonary arterial dilatation might help differentiate tumoral embolism from bland embolus. The distinction between tumoral and bland pulmonary thrombus is crucial, as it carries treatment and prognostic implications. Evaluation using endoscopic bronchial ultrasound and pulmonary artery catheter aspiration has also been mentioned as invasive diagnostic modalities [10-13].

Management and role of interventional radiology

Patients with symptomatic pulmonary embolism have high morbidity and mortality [14]. With advancements in minimally invasive endovascular treatment modalities, management of pulmonary embolism has witnessed a shift from systemic thrombolysis or surgical thromboarterectomy to catheter-directed thrombolysis, mechanical thrombectomy, or pharmaco-mechanical thrombectomy, combining mechanical thrombectomy with catheter-directed thrombolysis [15]. However, the management of PTE is still challenging due to indistinct imaging appearance and lack of adequate response to anticoagulation. Presently, there is a lack of dedicated guidelines for the management of PTE, and the data are limited to case reports and case series. However, in view of similar clinical presentation, management can be extrapolated from the guidelines for acute pulmonary embolism [16]. The American Heart Association (AHA) guidelines recommend mechanical thrombectomy with or without thrombolysis in patients with overt or incipient cardiopulmonary failure [16]. Besides restoring central pulmonary flow resulting in hemodynamic stability, aspiration thrombectomy can provide a tissue sample by retrieving the embolic material for histopathological evaluation. This is particularly important for management of patients with underlying cancer.

Computer-assisted vacuum thrombectomy (CAVT) using large-bore aspiration catheters has been seen to be safe and effective for intermediate- and high-risk pulmonary thromboembolism [17,18]. Compared to systemic thrombolysis and therapeutic anticoagulation, large-bore suction thrombectomy provides immediate, on-table clinical improvement while minimizing the risk of hemorrhagic adverse effects. Intra-procedural inability to obtain a significant amount of thrombus despite aggressive suction thrombectomy, difficulty in performing mechanical clot disruption secondary to adherence of thrombus to the vessel wall, and prevention of passage of the guide wire past the filling defect should raise suspicion of tumoral thrombus. In such cases, the clot sample should be sent for histopathological analysis to exclude an underlying neoplastic thrombus. Histopathological confirmation not only enables the clinician to tailor the medical management but also facilitates a management plan in cases of recurrence or suboptimal recanalization. In the latter case, tumoral embolism might need more aggressive measures like stent deployment to ensure vessel patency.

## Conclusions

The management of PTE involves a multidisciplinary approach, incorporating anticoagulation, systemic therapy for underlying malignancy, and supportive care measures. Systemic therapy, including chemotherapy, immunotherapy, or targeted therapy, should be tailored to the specific tumor type and stage with the goal of controlling tumor burden and reducing the risk of further embolic events. The prognosis of PTE is generally poor, reflecting the advanced stage of malignancy and the inherent thromboembolic risk associated with cancer. However, minimally invasive techniques like endovascular pulmonary thrombectomy can significantly decrease the morbidity and mortality associated with the disease.
